# A Multiplex Crystal Digital PCR for Detection of African Swine Fever Virus, Classical Swine Fever Virus, and Porcine Reproductive and Respiratory Syndrome Virus

**DOI:** 10.3389/fvets.2022.926881

**Published:** 2022-06-24

**Authors:** Kaichuang Shi, Yating Chen, Yanwen Yin, Feng Long, Shuping Feng, Huixin Liu, Sujie Qu, Hongbin Si

**Affiliations:** ^1^College of Animal Science and Technology, Guangxi University, Nanning, China; ^2^Guangxi Center for Animal Disease Control and Prevention, Nanning, China

**Keywords:** crystal digital PCR, African swine fever virus, classical swine fever virus, porcine reproductive and respiratory syndrome virus, detection of clinical sample

## Abstract

African swine fever (ASF), classical swine fever (CSF), and porcine reproductive and respiratory syndrome (PRRS) are highly infectious diseases of domestic pigs and wild boars. The co-infections of ASF virus (ASFV), CSF virus (CSFV), and PRRS virus (PRRSV) have been reported in different pig farms. Early differential detection and diagnosis of ASFV, CSFV, and PRRSV in the clinical samples is very important for the effective prevention and control of these diseases. A multiplex crystal digital PCR (dPCR) was developed for differential detection of ASFV, CSFV, and PRRSV in this study, targeting p72, 5' untranslated region (UTR), and ORF7 genes, respectively. The different reaction conditions were optimized, and the specificity, sensitivity, and repeatability of the assay were evaluated. The results showed that the multiplex crystal dPCR was able to accurately and differentially detect ASFV, CSFV, and PRRSV with a limit of detection of 4.69 × 10^−1^ copies/μl, respectively, and could not detect other porcine viruses, i.e., foot-and-mouth disease virus (FMDV), Senecavirus A (SVA), atypical porcine pestivirus (APPV), pseudorabies virus (PRV), porcine circovirus type 2 (PCV2), and porcine parvovirus (PPV). The assay showed excellent repeatability and reproducibility, with coefficients of variation (CV) of the intra- and inter-assay from 0.09 to 1.40%, and from 0.64 to 2.26%, respectively. The 289 clinical samples from different pig herds in Guangxi province, China, were tested by the multiplex crystal dPCR and a reference multiplex real-time quantitative RT-PCR (qRT-PCR) established previously in our laboratory. The positive rates of ASFV, CSFV, and PRRSV were 30.10, 13.49, and 22.49% by the multiplex crystal dPCR, and 24.57, 8.65, and 18.34% by the multiplex qRT-PCR, with coincidence rates of 94.66, 95.16, and 95.84%, respectively. The results indicated that the established multiplex crystal dPCR was a specific, sensitive, and accurate method for the detection and quantification of ASFV, CSFV, and PRRSV. This is the first report on the multiplex dPCR for detecting ASFV, CSFV, and PRRSV.

## Introduction

African swine fever virus (ASFV) is a double-stranded enveloped DNA virus which belongs to the genus *Asfivirus* of the family *Asfarviridae* (1), and causes ASF in domestic pigs and wild boars with the characterization of high fever, intensive lymphoid tissue damage, and extensive hemorrhage (2). Classical swine fever virus (CSFV) is a single-stranded, positive-sense RNA virus with an envelope and is a member of the *Pestivirus* genus of the family *Flaviviridae* (3), and causes CSF in pigs with the characterization of high fever and severe hemorrhage. Porcine reproductive and respiratory syndrome virus (PRRSV) is a single-stranded, positive-sense RNA virus with an envelope which belongs to the *Porarterivirus* genus of the family *Arteriviridae* (4), and causes abortion in pregnant sows, and respiratory disorders of all ages of pigs. ASF, CSF, and PRRS are notifiable diseases to the World Organization for Animal Health (OIE). The clinical manifestations and pathological damages of ASF, CSF, and PRRS are very similar and sometimes hard to be discriminated against in the field. Furthermore, ASF, CSF, and PRRS co-infections occur occasionally in pig herds (5–8), and this increases the difficulty of diagnosis only based on clinical signs, and needs to be diagnosed by laboratory assays. Therefore, an assay for rapid, accurate, and reliable detection of ASFV, CSFV, and PRRSV in the clinical samples is necessary to rapidly differentiate and diagnose these diseases.

Currently, polymerase chain reaction (PCR)/reverse transcription-PCR (RT-PCR) and real-time quantitative PCR (qPCR)/qRT-PCR as rapid, specific, and sensitive molecular techniques for viral detection are being widely used in many laboratories (9, 10). However, the conventional PCR/RT-PCR assays are unsuitable for high-throughput testing due to the requirement of gel analysis of the PCR products. The qPCR/qRT-PCR assays have been widely used due to their lower pollution probability, faster reaction speed, and higher sensitivity compared with the conventional PCR assays, but still have some limitations because the result depends on the relationship between cycle threshold (Ct) and standard calibration curve (11). Until now, some assays have been reported on differential detection of ASFV, CSFV, and PRRSV by multiplex RT-PCR (7, 12, 13) and multiplex qRT-PCR (8, 14–18).

Digital PCR (dPCR), a new emerging PCR technique, can quantify nucleic acid without reliance on external standards, standard curves, and Ct values (19, 20). Currently, the available commercial dPCR platforms are mainly dependent on two distinct approaches, namely chamber digital PCR (cdPCR) and droplet digital PCR (ddPCR) (21, 22). Compared with qPCR/qRT-PCR, dPCR shows the advantages of absolute quantification independent of calibration curves, lower sensitivity to PCR inhibitors, higher accuracy, reliability, and repeatability (23). Of the current commercial dPCR platforms, the Naica^TM^ (Stilla Technologies, Villejuif, France) is one of the most notable brands. The Naica^TM^ System, named as crystal digital PCR, combines the droplet partitions of ddPCR and the 2D array format of cdPCR (22, 24). The reaction mixture is transported to the inlet ports of the Sapphire chips, partitioned into droplet crystals, thermocycled on the Naica^TM^ Geode (a flat-block thermocycler), and further transferred onto the Naica^TM^ Prism3 (a fluorescence microscope) and imaged to reveal the amplified partitions. In addition, the Naica^TM^ System can perform multicolor multiplex dPCR based on a three-color or six-color detecting instrument (24). In this study, a three-color multiplex crystal dPCR assay based on the Naica^TM^ System was developed for differential detection of ASFV, CSFV, and PRRSV in a single reaction. This is the first report on the multiplex dPCR for detecting these pathogens.

## Materials and Methods

### Viral Strains and Clinical Tissue Samples

The 289 clinical samples from different pig herds, including tonsil, lymph nodes, liver, spleen, lung, kidney, and brain of each dead pig suspected ASF, CSF, and PRRS, were collected in Guangxi province, southern China, between January 2018 and March 2021. The collected samples were transported immediately at ≤ 4°C to our laboratory and stored at −70°C until used.

The vaccine strains of CSFV (C-strain), foot-and-mouth disease virus (FMDV, O/Mya98/XJ/2010 strain), and PRRSV (TJM-92 strain) were bought from the Tecon Animal Husbandry Biotechnology Co., Ltd, China, and pseudorabies virus (PRV, Bartha-K61 strain), porcine circovirus type 2 (PCV2, SX07 strain), and porcine parvovirus (PPV, WH-1 strain) were bought from China Animal Husbandry Industry Co., Ltd. The clinically positive samples of ASFV, Senecavirus A (SVA), and atypical porcine pestivirus (APPV) were provided by our laboratory. They were stored at −70°C until used.

### Primers and Probes

The ASFV p72 gene, CSFV 5' untranslated region (UTR), and PRRSV ORF7 gene were used as the targets of the multiplex crystal dPCR. The specific primers and probes were designed using Primer Express software (Version 3.0.1, Applied Biosystems, United States of America) and their detailed sequence is shown in Table 1.

**Table 1:** The primers and probes for detection of ASFV, CSFV, and PRRSV.

**Name**	**Sequence (5^**′**^-3^**′**^)**	**Length (bp)**	**Product size (bp)**
ASFV-p72-F	GGCGTATAAAAAGTCCAGGAAATTC	25	79
ASFV-p72-R	TTCGGCGAGCGCTTTATC	18	
ASFV-p72-P	FAM-TCACCAAATCCTTTTGCGATGCAAGCT-BHQ1	27	
CSFV-5′UTR-F	CCTGAGTACAGGACAGTCGTCAGT	24	72
CSFV-5′UTR-R	CCCTCGTCCACATAGCATCTC	21	
CSFV-5′UTR-P	VIC-TTCGACGTGAGCAGAAGCCCACC-BHQ1	23	
PRRSV-ORF7-F	GTTTGTGCTTGCTAGGCCG	19	178
PRRSV-ORF7-R	CTGCCACCCAACACGAGG	18	
PRRSV-ORF7-P	Cy5-ATTCTGGCCCCTGCCCACCACG -BHQ3	22	

### Preparation of Recombinant Standard Plasmids

The recombinant standard plasmids containing 79 bp of ASFV p72 gene, 72 bp of CSFV 5'UTR, and 178 bp of PRRSV ORF7 gene were constructed and named as p-ASFV, p-CSFV, and p-PRRSV as previously reported (8). The concentrations of all three plasmids were determined to be 4.69 × 10^9^ copies/μl, and stored at −20°C until used.

### Nucleic Acid Extraction and Reverse Transcription

The total DNA/RNA was extracted from all clinical samples using the Nucleic Acid Extraction & Purification Kit (DT6) (TIANLONG, China), and then reverse-transcribed to cDNA using the FastKing RT Kit (with gDNase) (TIANGEN, China). The extracted DNA was used for the detection of ASFV, and the cDNA was used for the detection of CSFV and PRRSV.

### Optimization for the Multiplex Crystal dPCR

The Naica^TM^ System (Stilla Technologies, Villejuif, France) was used to develop multiplex crystal dPCR. The 25 μl PCR system consisted of PerfeCta Multiplex qPCR ToughMix UNG (Quanta Biosciences, Gaithersburg, MD, United States of America), Fluorescein Sodium Salt (Apexbio Biotechnology, Beijing, China), which allows adequate imaging of all droplets for software analysis, primers, and probes of ASFV, CSFV and PRRSV, DNA/cDNA templates, and distilled water (Table 2). The reaction conditions, including the probe concentrations (from 200 to 400 nM), the primer concentrations (from 400 to 900 nM), and annealing temperatures (from 56 to 61°C) were optimized. No template control (NTC) was used as a negative control. The PCR was performed with an initial step at 95°C for 5 min; followed by 45 cycles of 95°C 5 s, 59°C 30 s, 72°C 30 s, and 72°C for 5 min as a final step.

**Table 2:** The reaction system of the multiplex crystal dPCR.

**Reagents**	**Multiplex crystal dPCR**		**Multiplex qRT-PCR**	
	**Volume (μl)**	**Final concentration (nM)**	**Volume (μl)**	**Final concentration (nM)**
Premix Ex Taq (Probe qPCR) (2 ×)	/	/	12.5	1 ×
PerfeCta Multiplex qPCR ToughMix (2 ×)	12.5	1 ×	/	/
Fluorescein Sodium Salt (1 μM)	2.5	100	/	/
ASFV-p72-F (25 μM)	0.9	900	0.4	400
ASFV-p72-R (25 μM)	0.9	900	0.4	400
ASFV-p72-P (25 μM)	0.3	300	0.4	400
CSFV-5'UTR-F (25 μM)	0.8	800	0.4	400
CSFV-5'UTR-R (25 μM)	0.8	800	0.4	400
CSFV-5'UTR-P (25 μM)	0.2	200	0.5	500
PRRSV-ORF7-F (25 μM)	0.9	900	0.3	300
PRRSV-ORF7-R (25 μM)	0.9	900	0.3	300
PRRSV-ORF7-P (25 μM)	0.3	300	0.3	300
Template	2.5	/	2.5	/
RNase Free H_2_O	Up to 25	/	Up to 25	/

The multiplex crystal dPCR process produces 25,000–30,000 analyzable droplets, equivalent to 5 logs of the theoretical dynamic, ranging from 0.2 copies/μl to 20,000 copies/μl. The Naica™ Prism3 (Stilla Technologies, Villejuif, France) automatically determined the absolute concentration of the template.

### Process of the Crystal dPCR

The Naica^TM^ System (Stilla Technologies, Villejuif, France) was used as the multiplex crystal dPCR amplification platform. The reaction system and amplification cycle, including Sapphire chips (Stilla Technologies, France) preparation, droplet generation, amplification, and fluorescence imaging and analysis were performed briefly as follows.

#### First, Preparation of the Sapphire Chips

Reaction mixtures of a total volume of 25 μl were prepared according to optimization of the reaction conditions. Each Sapphire chip was pipetted into each of the 4 inlet ports with 25 μl reaction mixture, primed with an emulsion oil, and sealed with removable Luer caps.

#### Second, Partition and PCR Amplification

The prepared Sapphire chips were placed onto the Naica^TM^ Geode (Stilla Technologies, France) and then launched the combined partition and PCR program. The PCR amplifications were carried out at 95°C for 5 min; followed by 45 cycles of 95°C 5 s, 59°C 30 s, 72°C 30 s; and 72°C for 5 min as a final step.

#### Third, Acquisition of 3-Color Fluorescence Images and Analysis of the Droplet Crystals

After amplification, the chips were shifted into the Naica^TM^ Prism3 (Stilla Technologies, France), imaged fluorescence for each droplet crystal with 3 high-resolution images, and discriminated the positive and negative droplets using the Crystal Miner software package (Stilla Technologies, France). The Naica^TM^ Prism3 automatically reported each sample's absolute concentration.

### Analysis of the Specificity, Sensitivity, and Repeatability of the Multiplex Crystal dPCR

The DNA/cDNA of ASFV, CSFV, PRRSV, FMDV, SVA, APPV, PRV, PCV2, and PPV, which were common viruses found in Chinese pig herds, were used to validate the specificity of the established multiplex crystal dPCR.

The 10-fold serial dilution of p-ASFV, p-CSFV, and p-PRRSV standard plasmids from 4.69 × 10^3^ copies/μl to 4.69 × 10^−1^ copies/μl were used to evaluate the sensitivity of the multiplex crystal dPCR, and the limit of detection (LOD) of each plasmid was determined.

The p-ASFV, p-CSFV, and p-PRRSV standard plasmids, with different concentrations of 4.69 × 10^3^, 4.69 × 10^2^, and 4.69 × 10^1^ copies/μl, were run in triplicate to determine the coefficient of variation (CV) of intra-assay for repeatability. The assay was run for 3 days to determine the CV of inter-assay for reproducibility of the established multiplex crystal dPCR.

### The Multiplex qRT-PCR Assay

The multiplex qRT-PCR was developed using the QuantStudio^TM^ 5 qPCR detection system (ABI, United States of America) in our laboratory as previously reported (8). The standard curves were drawn with standard plasmids in 10-fold serial dilutions. The concentrations of ASFV, CSFV, and PRRSV in each sample were calculated according to the Ct values of the standard curves. The 25 μl qRT-PCR system comprised 1 × Premix Ex Taq (Probe qPCR) (TaKaRa, China), 400 nM ASFV forward and reward primers, 400 nM ASFV probe, 400 nM CSFV forward and reward primers, 500 nM CSFV probe, 300 nM PRRSV forward and reward primers, and 300 nM PRRSV probe (Table 2). The amplifications were performed at 95°C for 2 min, and then, 40 cycles at 95°C for 5 s and 56°C for 34 s. All reactions were provided negative and positive controls, and each sample was determined in triplicate. The Ct values were generated by the QuantStudio^TM^ Design & Analysis Software (ABI, United States of America), and the sample with a Ct value ≤ 35 was considered positive.

### Detection of the Clinical Samples

The 289 clinical samples were tested by the established multiplex crystal dPCR and the reference multiplex qRT-PCR. No template as negative control and the standard plasmids as positive control were included in each reaction. The coincidence rate of these two assays was determined.

### Statistical Analysis

GraphPad Prism version 7.04 software (LA Jolla, California, United States of America) and SPSS version 26.0 software (IBM, United States of America) were used for statistical analysis of the linear regression and the coincidence rate.

## Results

### Optimization of the Parameters of the Multiplex Crystal dPCR

**Figure 1:**
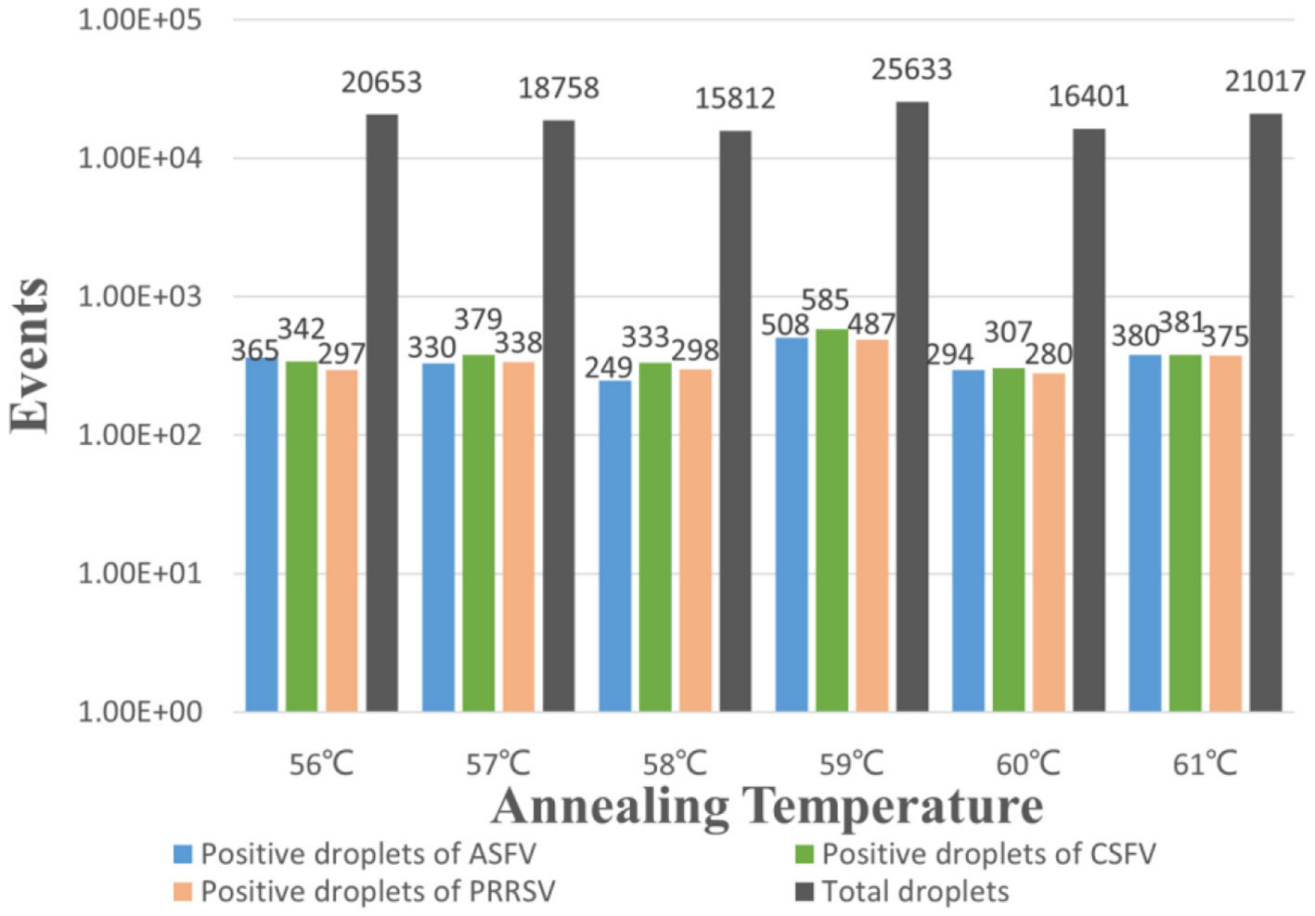
Optimization of the annealing temperature for the multiplex crystal dPCR. The bars showed the positive droplets and the total droplets with a gradient of 56, 57, 58, 59, 60 and 61°C.

**Figure 2:**
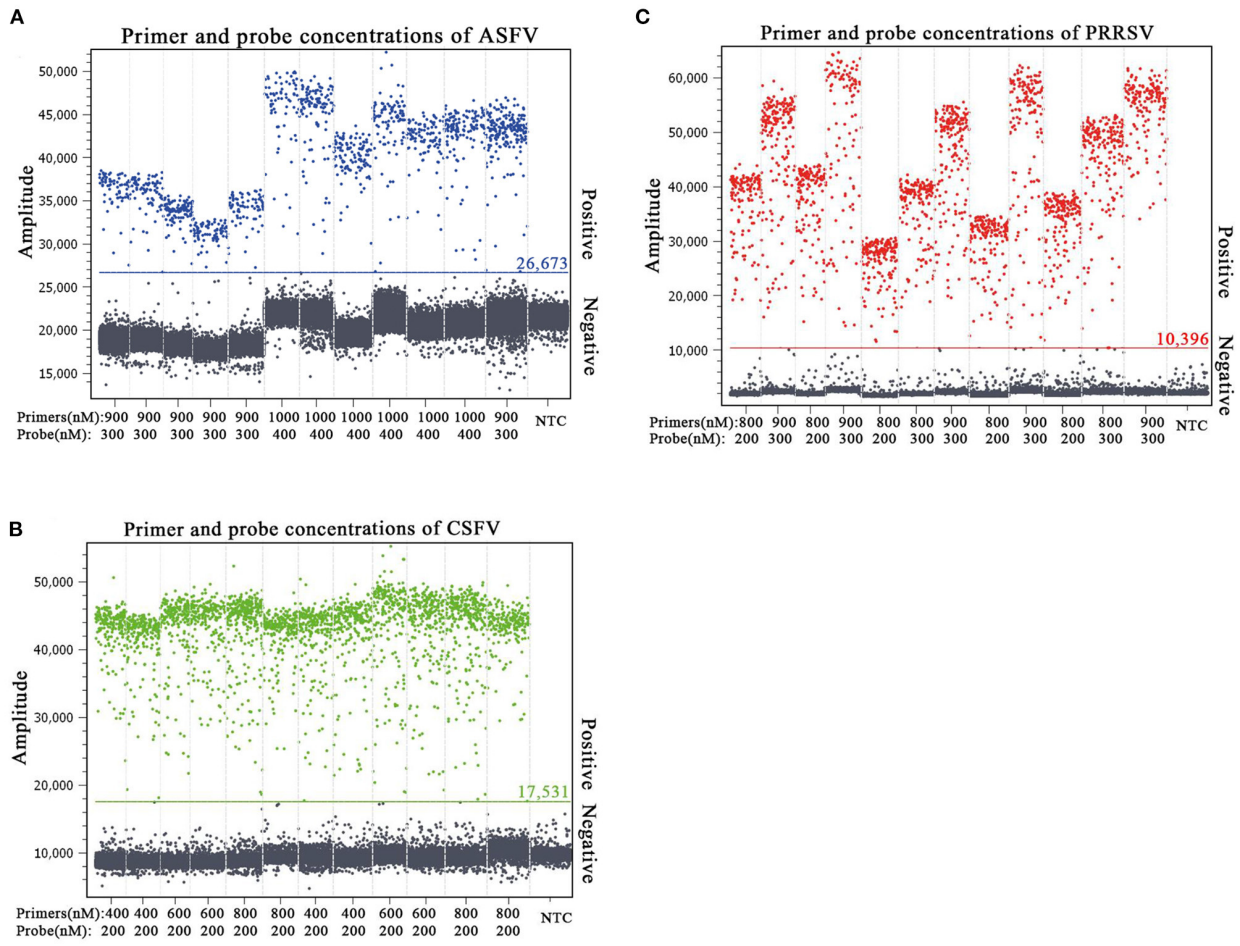
Determination of the optimal primer and probe concentrations of the multiplex crystal dPCR for detection of ASFV **(A)**, CSFV **(B)**, and PRRSV **(C)**. The fluorescence amplitudes of different combinations of primer and probe concentrations for the multiplex crystal dPCR. NTC: no template control.

The p-ASFV, p-CSFV, and p-PRRSV standard plasmids were used to optimize the optimal annealing temperature, primer concentration, and probe concentration of the multiplex crystal dPCR. The plasmids were 10-fold serially diluted and the mixture of 4.69 × 10^2^ copies/μl for each plasmid was used as the template for the multiplex crystal dPCR with annealing temperatures from 56 to 61°C. The result showed that the optimal annealing temperature was 59°C, which could generate the most total droplets and positive droplets (Figure 1). In addition, the probe concentrations and primer concentrations were also optimized by three standard plasmids with a mixture of 4.69 × 10^2^ copies/μl for each plasmid. The annealing temperature was fixed at 59°C, while the arrangement and combination of different concentrations of primers and probes were analyzed using the Crystal Miner software (Stilla Technologies, Villejuif, France) (Figures 2A–C). The concentration combinations with the largest fluorescence amplitude interval between negative (gray) and positive (color) with obvious boundaries were determined as the optimal primer concentrations and probe concentrations. The optimal primer and probe concentrations are listed in Table 2.

After optimization of the reaction conditions, the multiplex crystal dPCR was successfully developed. A total volume of 25 μl reaction mixtures contained 12.5 μl of PerfeCta Multiplex qPCR ToughMix UNG (2 ×) (Quanta Biosciences, Gaithersburg, MD, United States of America), 2.5 μl of Fluorescein Sodium Salt (1 μM) (Apexbio Biotechnology, Beijing, China), 0.9 μl of primers ASFV-p72-F/R each (25 μM), 0.3 μl of probe ASFV-p72-P (25 μM), 0.8 μl of primers CSFV-5'UTR-F/R each (25 μM), 0.2 μl of probe CSFV-5'UTR-P (25 μM), 0.9 μl of primers PRRSV-ORF7-F/R each (25 μM), 0.3 μl of probe PRRSV-ORF7-P (25 μM), 2.5 μl of DNA/cDNA template, and 1.5 μl of RNase free water (Table 2). The PCR amplifications were carried out as follows: 95°C for 5 min; 45 cycles of 95°C for 5 s, 59°C for 30 s, 72°C for 30 s; and a final step at 72°C for 5 min. After amplification, the absolute concentration of each sample was automatically reported by the Naica^TM^ System.

### Specificity and Repeatability of the Multiplex Crystal dPCR

**Figure 3:**
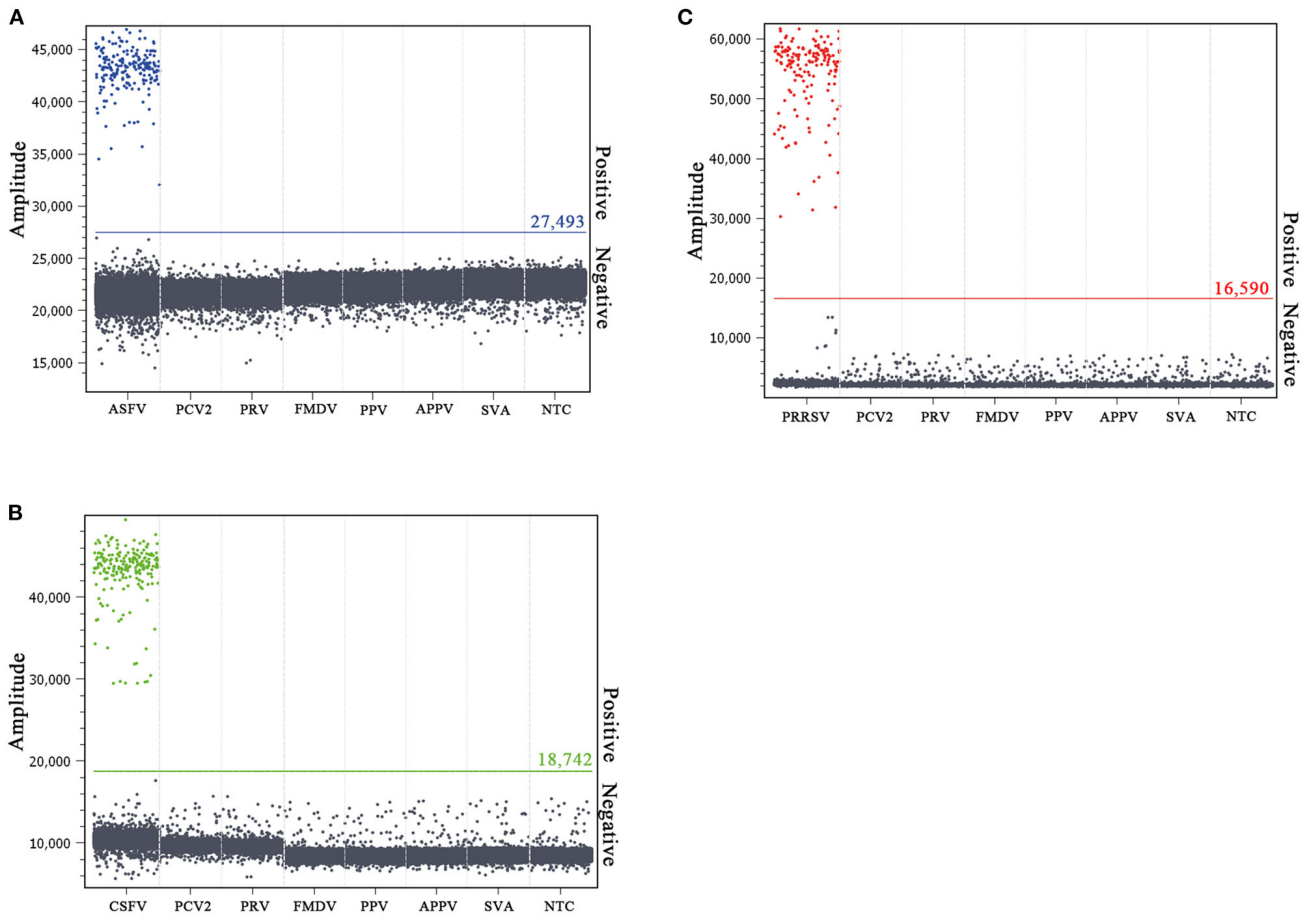
Specificity analysis of the multiplex crystal dPCR for detection of ASFV **(A)**, CSFV **(B)**, and PRRSV **(C)**. The fluorescence amplitudes of ASFV, CSFV, PRRSV, FMDV, SVA, APPV, PRV, PCV2, and PPV were showed. NTC: no template control.

The specificity of the multiplex crystal dPCR assay was evaluated using the DNA/cDNA of ASFV, CSFV, PRRSV, FMDV, SVA, APPV, PRV, PCV2, PPV, and negative control as templates. The results showed that fluorescence signals were obtained only from ASFV, CSFV, and PRRSV, and could not be obtained from FMDV, SVA, APPV, PRV, PCV2, PPV, and negative control (Figures 3A–C), indicating that the multiplex crystal dPCR assay was specific for the detection of ASFV, CSFV, and PRRSV.

Three concentrations of 4.69 × 10^3^, 4.69 × 10^2^, and 4.69 × 10^1^ copies/μl for each standard plasmid in the mixture were used as templates to evaluate the repeatability and reproducibility. The results showed that the CVs of intra-assay for repeatability were from 0.09 to 1.40%, and the CVs of inter-assay for reproducibility were from 0.64 to 2.26% (Table 3), indicating excellent repeatability and reproducibility of the established multiplex crystal dPCR.

**Table 3:** Analysis of the repeatability and reproducibility of the multiplex crystal dPCR.

**Standard plasmid**	**Concentration (copies/μL)**	**Intra-assay of repeatability**				**Inter-assay of reproducibility**			
		**Measured values (copies/μl)**			**CV (%)^**a**^**	**Measured values (copies/μl)**			**CV (%)**
p-ASFV	4.69 ×10^3^	3,963	3,867	3,962	1.40	3,931	4,019	3,901	1.55
	4.69 ×10^2^	448.9	450.4	450.7	0.21	450	441	457.8	1.91
	4.69 ×10^1^	44.9	45.1	44.9	0.27	44.9	47	45.9	2.20
p-CSFV	4.69 ×10^3^	4,447	4,455	4,450	0.09	4,451	4,507	4,484	0.64
	4.69 ×10^2^	478.7	477.1	477.5	0.17	477.8	469	482.8	1.47
	4.69 ×10^1^	48.5	48.8	48.7	0.31	48.7	50	50.2	1.64
p-PRRSV	4.69 ×10^3^	3,908	3,900	3,903	0.10	3,903	3,832	3,920	1.20
	4.69 ×10^2^	445.5	444.8	444.1	0.16	445	450	451.8	0.80
	4.69 ×10^1^	46.4	46.2	47	0.90	46.5	47.7	45.6	2.26

^a^*CV stands for coefficient of variation*.

### Comparison of the Sensitivity and Standard Curves of the Multiplex Crystal dPCR and the Multiplex qRT-PCR

The sensitivity test using standard plasmids from 4.69 × 10^3^ copies/μl to 4.69 × 10^−1^ copies/μl showed that the limit of detection (LOD) of p-ASFV, p-CSFV, and p-PRRSV was 4.69 × 10^−1^ copies/μl by the multiplex crystal dPCR, and was 4.69 × 10^0^ copies/μl by the multiplex qRT-PCR, indicating that the multiplex crystal dPCR was ten times higher than the multiplex qRT-PCR (Table 4).

**Table 4:** Sensitivity comparison of the multiplex crystal dPCR and the multiplex qRT-PCR.

**Concentration (copies/μL)**	**Crystal dPCR (Mean copies/μl)**			**qRT-PCR (Mean Ct**^**a**^ **value)**		
	**ASFV**	**CSFV**	**PRRSV**	**ASFV**	**CSFV**	**PRRSV**
4.69 ×10^3^	3,967	4,447	3,908	23.002	24.303	23.643
4.69 ×10^2^	450.7	477.5	445.5	26.113	27.975	27.043
4.69 ×10^1^	45.1	48.5	47	29.539	31.322	30.710
4.69 ×10^0^	3.99	4.16	4.01	32.872	34.014	33.814
4.69 ×10^−1^	0.43	0.43	0.43	36.481	38.768	36.723
NTC^b^	ND^c^	ND	ND	ND	ND	ND

^a^*Ct stands for cycle threshold. ^b^NTC stands for no template control. ^c^ND stands for not detected. The limit of detection (LOD) was 4.69 × 10^−1^ copies/μL for the multiplex crystal dPCR, and was 4.69 × 10^0^ copies/μL for the multiplex qRT-PCR*.

**Figure 4:**
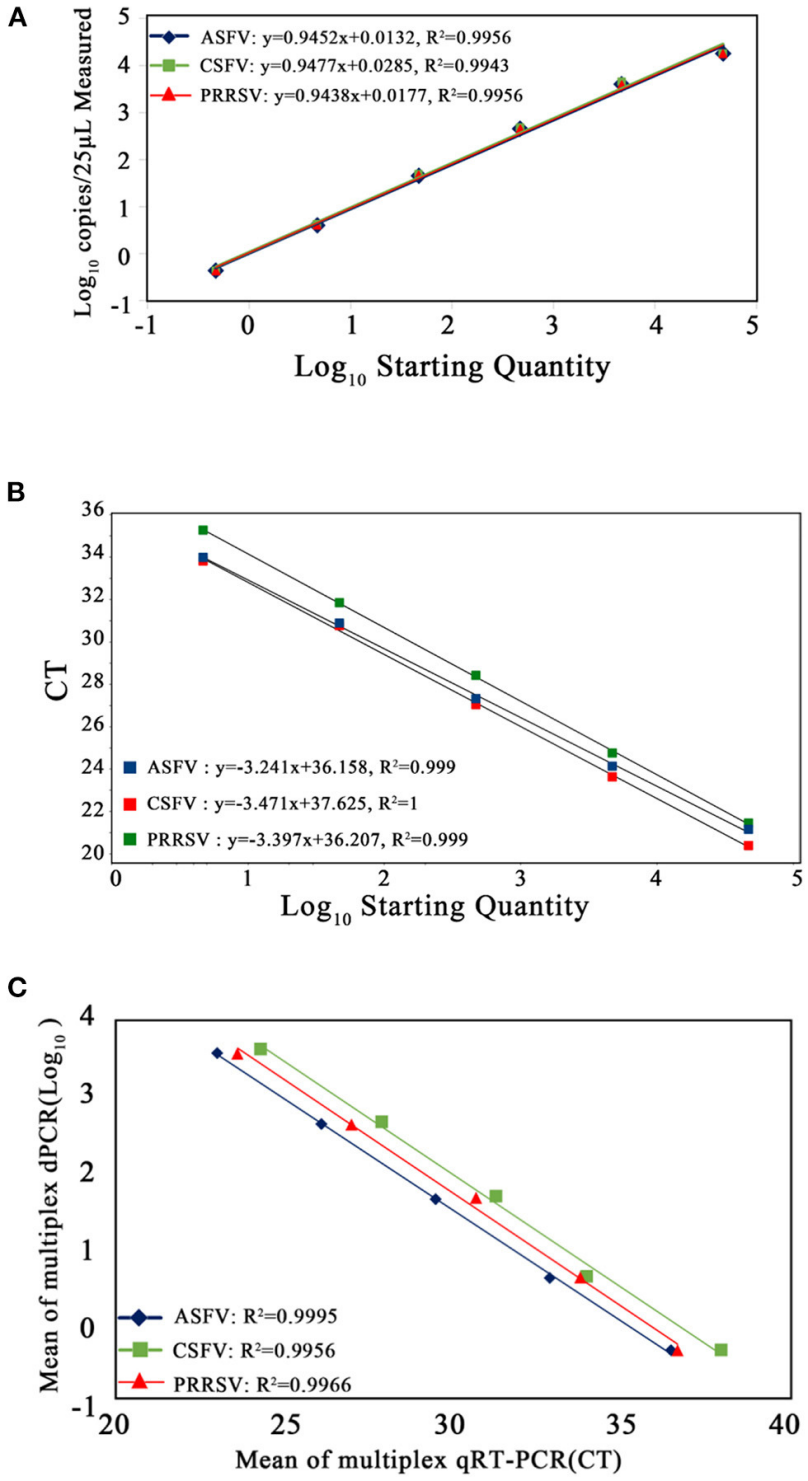
Standard curves of the multiplex crystal dPCR **(A)**, the multiplex qRT-PCR **(B)**, and the correlation between these two methods **(C)**. The 10-fold serially diluted p-ASFV, p-CSFV and p-PRRSV standard plasmids from 4.69 × 10^4^ to 4.69 × 10^−1^ copies/μl were used to generate the standard curves. The correlation between these two methods was acquired by plotting the logarithm of absolute measured values of the multiplex crystal dPCR against the logarithm of cycle threshold (Ct) values of the multiplex qRT-PCR.

The p-ASFV, p-CSFV, and p-PRRSV plasmids from 4.69 × 10^4^ copies/μl to 4.69 × 10^−1^ copies/μl were used to generate the standard curves. The multiplex crystal dPCR (*R*^2^ was 0.9956 for ASFV, 0.9943 for CSFV, and 0.9956 for PRRSV) and the multiplex qRT-PCR (*R*^2^ was 0.999 for ASFV, 1 for CSFV, and 0.999 for PRRSV) exhibited excellent linearity (Figures 4A,B). The slope values of the multiplex crystal dPCR were 0.9452 for ASFV, 0.9477 for CSFV, and 0.9438 for PRRSV, while the slope values of the multiplex qRT-PCR were −3.241 for ASFV, −3.471 for CSFV, and −3.397 for PRRSV. The Pearson correlation coefficients between the multiplex crystal dPCR and the multiplex qRT-PCR were 0.9995 for ASFV, 0.9956 for CSFV, and 0.9966 for PRRSV (Figure 4C), indicating a positive association between these two methods.

### Evaluation of the Crystal dPCR Assay by Clinical Samples

**Figure 5:**
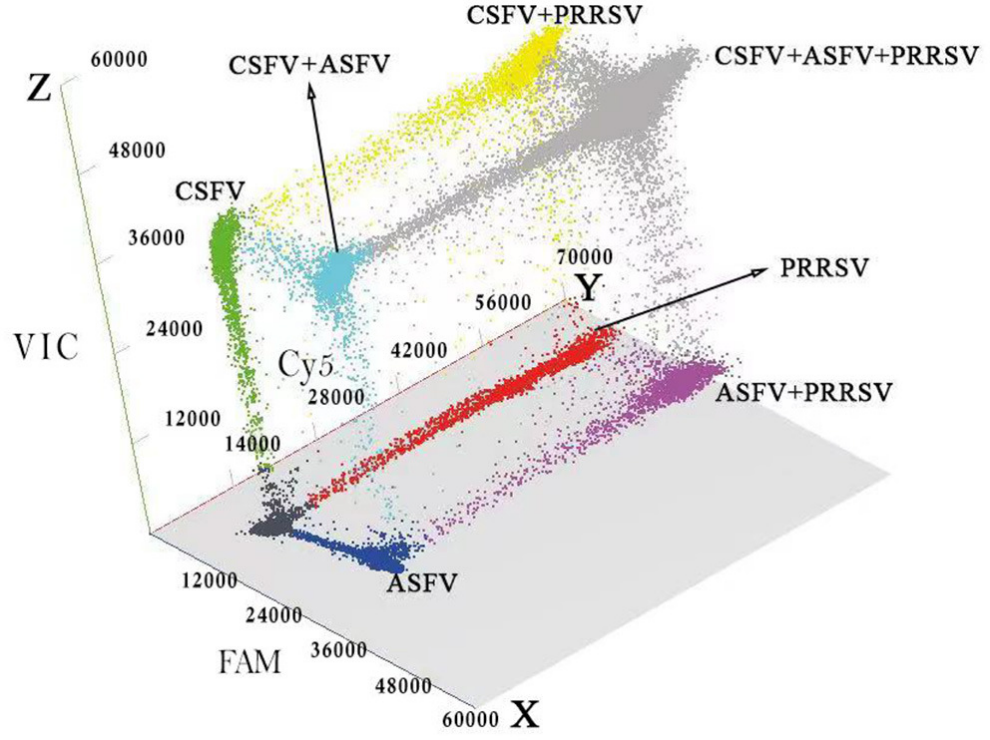
Detection results of the clinical samples. The 3D scatterplots of fluorescence intensities were acquired in the Blue (x axis), Red (y axis) and Green (z axis) acquisition channels.

The 289 clinical samples were tested by the developed multiplex crystal dPCR and the multiplex qRT-PCR. The results by the multiplex crystal dPCR showed that 30.10, 13.49, and 22.49% samples were positive for ASFV, CSFV, and PRRSV, while 5.88, 6.57, 5.88, and 2.77% samples were co-infected with ASFV + CSFV, ASFV + PRRSV, CSFV + PRRSV, and ASFV + CSFV + PRRSV, respectively. The results by the multiplex qRT-PCR showed that 24.57, 8.65, and 18.34% samples were positive for ASFV, CSFV, and PRRSV, while 2.77, 2.08, 2.42, and 0.35% samples were co-infected with ASFV + CSFV, ASFV + PRRSV, CSFV + PRRSV, and ASFV + CSFV + PRRSV, respectively (Table 5). The co-infections in clinical samples from this study are shown in a 3D dot plot in Figure 5 and displays data of a given sample using three-dimensional scatterplots allowing immediate visualization. The results indicated that the positive rates of the multiplex crystal dPCR were higher than those of the multiplex qRT-PCR, and their coincidence rates of ASFV, CSFV, and PRRSV were 94.46, 95.16, and 95.84%, respectively (Table 6).

**Table 5:** Detection results of the clinical samples by the multiplex crystal dPCR and the multiplex qRT-PCR.

**Date**	**Number**	**ASFV**		**CSFV**		**PRRSV**		**ASFV** **+** **CSFV**^**a**^		**ASFV** **+** **PRRSV**^**b**^		**CSFV** **+** **PRRSV**^**c**^		**ASFV** **+** **CSFV** **+** **PRRSV**^**d**^	
		**dPCR^**e**^**	**qPCR^**f**^**	**dPCR**	**qPCR**	**dPCR**	**qPCR**	**dPCR**	**qPCR**	**dPCR**	**qPCR**	**dPCR**	**qPCR**	**dPCR**	**qPCR**
2018	80	2	1	10	8	29	24	2	1	3	1	8	5	1	0
2019	80	29	24	12	9	10	9	2	1	7	4	3	2	2	1
2020	83	39	32	15	8	20	17	11	6	6	1	4	0	3	0
2021	46	17	14	2	0	6	3	2	0	3	0	2	0	2	0
Total	289	87	71	39	25	65	53	17	8	19	6	17	7	8	1
Positive rate (%)		30.10	24.57	13.49	8.65	22.49	18.34	5.88	2.77	6.57	2.08	5.88	2.42	2.77	0.35

^a^*ASFV + CSFV stands for co-infection of ASFV and CSFV. ^b^ASFV + PRRSV stands for co-infection of ASFV and PRRSV. ^c^CSFV + PRRSV stands for co-infection of CSFV and PRRSV. ^d^ASFV + CSFV + PRRSV stands for co-infection of ASFV, CSFV, and PRRSV. ^e^dPCR stands for detection results by the multiplex crystal dPCR. ^f^qPCR stands for detection results by the multiplex qRT-PCR*.

**Table 6:** Agreements between the multiplex crystal dPCR and the multiplex qRT-PCR.

**Detection method**	**Detection results (positive samples/total samples)**		
	**ASFV**	**CSFV**	**PRRSV**
Multiplex crystal dPCR	87/289	39/289	65/289
Multiplex qRT-PCR	71/289	25/289	53/289
Coincidence rates	94.46%	95.16%	95.84%
Kappa	0.86	0.76	0.87

## Discussion

The dPCR can be considered to be a modified version of conventional PCR for absolutely quantifying nucleic acids using the same Taq polymerase, primers, and probes, but the dPCR provides higher sensitivity and precision, higher tolerance to inhibitors, and does not need standard curve (21–24). Therefore, the dPCR has been widely used in many laboratories. Nowadays, ASF, CSF, and PRRS are still epidemic in some countries, and ASFV, CSFV, and/or PRRSV co-infections are occasionally reported in some pig farms (5–8). The multiplex RT-PCR and multiplex qRT-PCR have been developed to differentially detect these pathogens (7, 8, 12–18). The novel single dPCR has also been developed to detect ASFV (25, 26) and PRRSV (27). However, no multiplex dPCR for differentially detecting these pathogens has been reported until now. Therefore, the multiplex crystal dPCR was tried to develop in this study.

Three pairs of specific primers and corresponding probes were designed for ASFV, CSFV, and PRRSV. After optimization of the reaction conditions, a multiplex crystal dPCR based on the Naica^TM^ System was successfully developed for differential detection of ASFV, CSFV, and PRRSV in one reaction. The assay showed high specificity, and could test only ASFV, CSFV, and PRRSV, but not other swine viruses. The assay also showed high sensitivity and had a LOD of 4.69 × 10^−1^ copies/μl for ASFV, CSFV, and PRRSV, which was ten times higher than that of the multiplex qRT-PCR. Finally, the assay showed high repeatability and reproducibility, and had CVs <2.26% for the intra- and inter-assay. The evaluation demonstrated that the positive signals of ASFV, CSFV, and PRRSV displayed distinct patterns in a 3D analysis system (Figure 5). In addition, the co-infection rates of ASFV, CSFV, and/or PRRSV by the multiplex crystal dPCR were higher than those by the multiplex qRT-PCR in this study (Table 5). The multiplex crystal dPCR has the obvious advantage to test low templates over the multiplex qRT-PCR (23), and some negative samples by the multiplex qRT-PCR due to very low templates were still showing positive by the multiplex crystal dPCR. Therefore, the results of the multiplex crystal dPCR were more reliable than those of the multiplex qRT-PCR. The higher efficiency of the multiplex crystal dPCR might be due to its lower sensitivity to PCR inhibitors (22). Nowadays, the crystal dPCR is still not scaled, like qRT-PCR platforms, for mass screening due to the high cost (21–24). The multiplex crystal dPCR in this study could detect three viruses in one reaction, which could reduce reagent cost, handling time, and workforce while compared with the singleplex dPCR and the multiplex qRT-PCR (23, 24, 28, 29). According to preliminary calculation, it costs about US $17.67 to detect a sample by the singleplex dPCR, US $7.86 to detect a sample by the multiplex dPCR, and US $3.83 to detect a sample by the multiplex qRT-PCR, which indicated that the cost of the multiplex dPCR was much lower than that of the singleplex dPCR, although it was still higher than that of the multiplex qRT-PCR. Therefore, the developed multiplex crystal dPCR could be used for high-throughput detection of multiple infections in clinical samples, especially for the clinical samples with a very low concentration of targeted viruses.

Since China is the largest pig breeding country in the world, it is very important to prevent and control ASF, CSF, and PRRS. After first being identified in China in August 2018 (30), ASF spread rapidly all over the country (31). To date, genotype I and genotype II ASFV strains, as well as the gene-deleted and wild-type ASFV strains, have been reported in China (14, 32–34). In China, CSF was first recorded in 1925 and still occurs occasionally in some pig herds nowadays even if the attenuated C-strain vaccine, which has been verified to be very effective, has been widely used since the 1950 s (35, 36). PRRS was first confirmed in China in 1996, and nowadays, both genotype I and genotype II strains are prevalent in many pig herds in China (37, 38). ASFV, CSFV, and PRRSV co-infections have been reported in some pig farms (5–8, 17). In this study, 289 clinical samples, collected in Guangxi province, southern China, between January 2018 and March 2021, were detected by the developed multiplex crystal dPCR, and 30.10, 13.49, and 22.49% samples were positive for ASFV, CSFV, and PRRSV, and the ASFV + CSFV, ASFV + PRRSV, CSFV + PRRSV, and ASFV + CSFV + PRRSV co-infection rates were 5.88, 6.57, 5.88, and 2.77%. These results confirmed that co-infections of ASFV, CSFV, and PRRSV were still common in southern China and the developed crystal dPCR is a useful tool for differential detection of multiple viruses. Especially, the multiplex crystal dPCR can detect a very low concentration of pathogens, and this is very important for ASFV, CSFV, and PRRSV in the early stage of infection, which helps to accurately identify, strictly restrict, and timely remove the infected pigs. Take ASFV as an example. The low virulence ASFV strains and the gene-deleted ASFV strains have been reported in China. The infected pigs rarely showed typical symptoms but excreted the virus intermittently for a long time (32, 33). The established multiplex crystal dPCR in this study was used to test the clinical samples, and many pigs in the early stage or persistent stage were accurately identified, and have been disposed in time and avoided huge losses.

In conclusion, an accurate and sensitive multiplex crystal dPCR is developed for differential detection and quantification of ASFV, CSFV, and PRRSV with a high degree of specificity and repeatability in this study. The assay had high sensitivity with a LOD of 4.69 × 10^−1^ copies/μl for three pathogens and was especially suitable for the detection of a low concentration of virus in the sample. Thus, this specific, sensitive, and accurate multiplex crystal dPCR is a valuable tool to differentially detect ASFV, CSFV, and PRRSV. To our knowledge, this is the first report to develop a multiplex crystal dPCR for differential detection and absolute quantification of ASFV, CSFV, and PRRSV.

## Data Availability Statement

The original contributions presented in the study are included in the article/supplementary material, further inquiries can be directed to the corresponding author/s.

## Ethics Statement

Written informed consent was obtained by the owners of the animals to use the clinical samples in this study. No live animal was handled, so no ethical approval was required for the study.

## Author Contributions

KS contributed to the experiment design, data analysis, and manuscript revision. YC contributed to study design, doing experiments, data collection, and manuscript drafting. YY, FL, SF, HL, and SQ contributed to clinical sample collection, doing experiments, and data analysis. HS contributed to laboratory supervision and manuscript editing. The submitted manuscript has been read and approved by all authors.

## Funding

This study was supported by the Key Research & Development Program (AB21238003), the Science & Technology Major Project (AA17204057) of Guangxi Science & Technology Bureau, China, and the Agricultural Science & Technology Program (Z202031, Z201954) of Guangxi Agricultural & Rural Bureau, China.

## Conflict of Interest

The authors declare that the research was conducted in the absence of any commercial or financial relationships that could be construed as a potential conflict of interest.

## Publisher's Note

All claims expressed in this article are solely those of the authors and do not necessarily represent those of their affiliated organizations, or those of the publisher, the editors and the reviewers. Any product that may be evaluated in this article, or claim that may be made by its manufacturer, is not guaranteed or endorsed by the publisher.
